# A short elective supports the attitudes of medicine and pharmacy students towards interprofessional learning: a pre-post design

**DOI:** 10.1186/s12909-025-06988-0

**Published:** 2025-03-18

**Authors:** Serdar Yilmaz, Martina Hahn, Sibylle C. Roll, Christiane Muth, Marjan van den Akker

**Affiliations:** 1 Institute of General Practice, Goethe University, Theodor-Stern-Kai 7, 60590 Frankfurt am Main, Germany; 2 Department of Mental Health, varisano Klinikum Frankfurt Hoechst, Gotenstraße 6-8, 65929 Frankfurt am Main, Germany; 3 Department of Psychiatry, Psychosomatics and Psychotherapy, University Hospital Frankfurt-Goethe University, Heinrich-Hoffmann-Str. 10, 60528 Frankfurt am Main, Germany; 4 Department of Public Health and Primary Care, KU Leuven, Leuven, Belgium; 5 Department of Family Medicine, School CAPHRI, Maastricht University, Maastricht, The Netherlands; 6 Faculty of Pharmacy, Department of Pharmacology and Clinical Pharmacy, Philipps University Marburg, Karl-von-Frisch-Str. 2, 35043 Marburg, Germany; 7 General Practice and Family Medicine, Medical School East-Westphalia, Bielefeld University, Morgenbreede 1, 33615 Bielefeld, Germany

**Keywords:** Medical education, Interprofessional learning, Attitude of health personnel, Students (medicine and pharmacy)

## Abstract

**Introduction:**

With increasing medical and pharmaceutical challenges, the importance of interprofessional working and education (IPE) is growing. The expected benefits of interprofessional collaboration (IPC) of physicians and pharmaceutical staff and the existing gap in their practical experience support this burgeoning importance of IPE. To date, evidence on how IPE can contribute to students’ attitudes on IPC is scarce.

**Purpose:**

This study aimed to evaluate whether an interprofessional educational intervention could lead to an improved attitude towards interprofessional collaboration.

**Method:**

25 medical students and 37 pharmacy students participated in a non-controlled exploratory before-after study. To assess attitudes towards IPE, students filled out the SPICE-2D questionnaire, which consists of three domains and ten items. Data was analyzed descriptively and using paired t-tests to test mean differences between the two measurements on domain and item level.

**Result:**

In total, 31 participants completed both pre- and post-surveys. We found significant improvements across all three domains of the SPICE-2D questionnaire. On item level we found significant improvements in seven out of ten items. No significant group differences were found (e.g. gender or study course).

**Conclusion:**

Results show that the elective with a focus on IPE had a positive impact on the attitudes of medical and pharmacy students about interprofessionalism and collaboration. Findings suggest and confirm that standardized and structured IPE can positively improve attitudes. Further studies are recommended to validate these findings, especially with bigger sample sizes and long-term effects.

## Introduction

With increasing medical and pharmaceutical challenges, the importance of interprofessional working and education (IPE) is growing. Organizations like the World Health Organization (WHO) and the Interprofessional Education Collaborative (IPEC) are expanding their work with research on IPE. Not only are they defining what IPE is, they are also emphasizing its importance to the healthcare system and patient care.

“Interprofessional education occurs when students from two or more professions learn about, from, and with each other to enable effective collaboration and improve health outcomes. Once students understand how to work interprofessionally, they are ready to enter the workplace as members of the collaborative practice team. This is a key step in moving health systems from fragmentation to a position of strength” [1].

Previous studies on the effects of interprofessional learning of pharmacy and medical students were promising. A study with pharmacy students and postgraduate family medicine residents evaluated the effect of interprofessional work and education and showed few significant pre-post improvements regarding agreement towards interprofessional work and team-based practice, understanding one’s role and responsibility in the collaborative practice and being confident that patient outcomes improve from collaborative practice. Also narrative comments about interprofessional work and practice were positive [2]. Nwaesei et al. focused on developing a multimodal, structured, interprofessional and experience-based education for medical and pharmacy students. Pre-post analyses with the Student Perceptions of Physician-Pharmacist Interprofessional Clinical Education (SPICE-2D) questionnaire showed that attitudes towards interprofessional work and education, towards the roles of different health professions in interdisciplinary teams, and towards the expected outcomes of collaborative work for patients improved significantly [3]. In a study conducted in 2014 by Zorek et al., medical and pharmacy students took part in a preplanned IPE experience and filled out the SPICE questionnaire before and after participation. Results showed significant improvements in all three domains [4].

In order to improve pharmaceutical care, it is important to raise the awareness of physicians about the benefits of collaboration with pharmacists [5]. Zielińska-Tomczak et al. note that interactions between physicians and pharmacists are limited to issues like prescription errors, drug availability or drug dosage; they also point out these groups’ lack of collaboration rules, respect and trust for one another. The authors indicate that IPE could help to develop communication skills and to build a professional relationship between physicians and pharmacists [6].

Since 2019 we have offered an elective “Managing Multimorbidity and Polypharmacy” for medicine and pharmacy students at Goethe University Frankfurt (Germany). One of the aims of this elective is to improve interprofessional working in the long term by impacting students’ attitude towards interprofessional working of future medical and pharmaceutical staff.

There have been few studies on the effect s of interprofessional education on attitudes towards interprofessional working. The majority of studies so far have focused on development and validation on questionnaires concerning this topic [7–9]. Despite previous studies, significant gaps remain. There is limited longitudinal research examining the impact of IPE interventions with medical and pharmacy students, although these two professions have a large overlap in their later work fields. Furthermore, few studies have used a validated questionnaire to assess the impact of these IPE interventions on students’ attitudes and perceptions. This study aimed to evaluate whether or not an interprofessional educational intervention leads to an improved attitude towards interprofessional collaboration. Differences between female and male students, as well as differences between medical students and pharmacy students, were explored.

## Methods

### Design

The current study was conducted prospectively and featured a noncontrolled before-after exploratory design. Primary outcomes were the students’ attitudes towards interprofessional collaboration as assessed by the SPICE-2D questionnaire.

### Participants and setting

The participants who took part in the elective were medical and pharmaceutical students in advanced course of study. The study of medicine at Goethe University Frankfurt is divided into two parts (preclinical and clinical). In the first part, the studies focus on foundational courses like anatomy, chemistry and physiology. The clinical phase then focuses on practical learning and application in various medical areas. The study of pharmacy is also divided into preclinical and clinical phases. The first phase covers basic subjects, like chemistry, biology and physics. The second, or clinical, phase then focuses on pharmaceutical practice, drug science and pharmacology. Medical as well as pharmacy students were allowed into the elective, from the clinical study phase onwards, which corresponds to year 4 of their studies. The participants were recruited after they had completed registration for the interprofessional elective. They were informed and instructed via e-mail, and questionnaires were handed out during the first session. These student participants received the questionnaire for the follow-up assessment via mail after finishing the elective. Participation was on a voluntary basis.

### Intervention

The elective was designed by three members from the medical and pharmaceutical faculties of our university and relied on their expertise and experiences. It is divided into two parts and taught for the most part in the classroom with mandatory physical attendance. The first part consists of four sessions. Of those, three sessions treat the concepts of multimorbidity and polypharmacy, pharmacoepidemiology, adherence, and offer case examples. In those three sessions, a variety of teaching methods (presentations, plenary discussions, small group discussions, and small-group work) were combined for use. There is one training session held with simulation patients with three different roles assigned. All of the students have to talk to at least one simulation patient. Following the practical phase, a plenary discussion is held, with a focus on patient communication, medication use, and potential drug interactions. The second part of the elective consists of one online session, and two physical visits to different hospitals (a geriatric ward and a mental health department) where students meet selected inpatients, and one visit to the hospital pharmacy located on the university campus. The elective is taught by involving instructors from various professional backgrounds, including an epidemiologist, a clinical pharmacist together with a psychiatrist, a physician for clinical pharmacology, a pharmacist and an assistant medical director. In all class meetings, students experience interprofessional learning, through discussion and assignments in mixed small groups. Students are only allowed to take part in the second part of the elective if they have completed the first part. During the Covid-19 pandemic, the elective was offered in a digital format.

### Questionnaire

The self-administered questionnaire used in this survey is a German version of the Student Perception of Physician Pharmacist Interprofessional Clinical Education (SPICE-2D).

We used this questionnaire to assess the students‘ views of interdisciplinary collaboration both before and after the elective class. Participants filled out the questionnaire prior to the start of the elective and right after course completion.

The original SPICE questionnaire was developed in 2013 and was refined and validated again in 2016 [7, 9]. It assesses the attitude of medical and pharmaceutical students towards interprofessional studies and work, using item statements like “Working with students of another discipline enhances my education”. Three team members of the Institute of General Practice of Goethe University Frankfurt with advanced skills in English and German translated the refined and validated SPICE-2 questionnaire into German. For the translation of the English version of the questionnaire, a translation method similar to Eremenco et al. was used [10]. First two team members independently translated the English version into German and discussed dissimilarities until they agreed on the formulation. A third team member, who was unaware of the original version, then performed a back-translation into English to ensure conceptual and linguistic equivalence. Differences and discrepancies between the original and back-translated versions were discussed and resolved collaboratively by the team members who were involved in the translation process.

The questionnaire consistsed of ten items in three domains: Interprofessional Teamwork and Team-based Practice (T); Roles/responsibilities for Collaborative Practice (R); Patient Outcomes from Collaborative Practice (O). Domain 1 had four items; the other two domains each consisted of three items. Each item assessed attitude on a five-point Likert scale, ranging from 1 (“do not agree at all”) to 5 (“completely agree”). Domain scores were calculated as the sum of the individual items and divided by the number of items of each domain. For each domain and each item, the mean score, standard deviation, median score and IQR were calculated.

### Data collection

The survey was available as a paper-pencil or digital survey. We collected data at baseline before the start of the elective and at follow-up time after participation in the elective, during five consecutive executions of the elective between Spring 2021 and Spring 2023. Both at baseline and at follow-up, students were asked to fill out the SPICE-2D questionnaire. The questionnaire was distributed and collected via e-mail or in the paper-based format. In addition to the ten items, participants were asked questions on their age, sex, semester and study. Filling out the questionnaire took about 5–10 min. To allow linkage of baseline and follow-up data, participants were asked to create a unique five-digit pseudonym consisting of the first letter of their mother’s surname, the first letter of their course of study (pharmacy or medicine), their mother’s date of birth in numbers and the first letter of their own place of birth. This method of pseudonymization uses information pieces, which are not easily accessible.

### Data analysis

The data was analyzed using SPSS 28 software. We looked at the item characteristics and checked whether the requirements of the inference statistics were met and whether or not there was any severe violation of normal distribution. For this study, we calculated the average scores of each item and each domain at baseline and follow-up. Score differences were tested using a t-test for paired samples for the total population. We used the independent samples t-test in order to determine whether statistical parameters differed significantly between relevant subgroups (sex, study, baseline participation vs. baseline and follow-up).

Cronbach’s alpha was calculated for the three domains. For domain 1, Cronbach’s alpha was moderate: α = 0.63 (McDonald’s omega 0.63); for domain 2, Cronbach’s alpha was also moderate: α = 0.64 (McDonald’s omega 0.64); and for domain 3, Cronbach’s alpha was satisfactory: α = 0.71 (McDonald’s omega 0.73). In line with Pudritz et al. [8] we considered a reliability of at least 0.6 acceptable while the desired value was 0.7 or greater.

To assess selective non-response at follow-up, we compared the baseline data of those students that participated in both measurements with those who only responded at baseline, using the independent samples t-test.

## Results

By the summer semester of 2023, a total of 84 participants had taken the elective, while 63 (75%) students had filled out the baseline survey. Students who completed the elective were in semesters 5 to 11 (MD 7.0). The average age of the participants was 25.6 (SD 5.44) years; 58.7% of them were pharmacy students. 69.8% of the participants were female. 31 participants also took part in follow-up of the survey and their data were eligible for data analysis. There were ten medical and 21 pharmacy students who completed both surveys. Of those, eight participants were male and 23 were female. The average age of those completing both surveys was 25.3 (SD 4.47) years.

At baseline we found a relatively high mean score for “interprofessional teamwork and team-based practice”, with $\:\stackrel{-}{x}$=4.64 on a five-point scale. Mean score in “roles/responsibilities for collaborative practice” was moderate, with $\:\stackrel{-}{x}$=3.40, and somewhat high for “patient outcomes from collaborative practice”, with $\:\stackrel{-}{x}$=4.08.

The three domains’ scores and scores at baseline and follow-up are presented in Table 1. The mean score in “interprofessional teamwork and team-based practice” rose to 4.77 after intervention, which was a significant mean improvement. The mean score in “roles/responsibilities for collaborative practice” significantly rose, to 3.88, whereas the mean score in “patient outcomes from collaborative practice” improved to 4.57.

**Table 1:** Results of the pairwise t-test for the three domains, *N* = 31

Domain	M T0 (SD)	M T1 (SD)	*P* *
Interprofessional teamwork and team-based practice (T)	4.64 (0.392)	4.77 (0.338)	*
Roles/responsibilities for collaborative practice (R)	3.40 (0.629)	3.88 (0.691)	***
Patient outcomes from collaborative practice (O)	4.08 (0.703)	4.57 (0.465)	***

M: mean score; SD: standard deviation; T0: baseline measurement; T1: measurement after completing the elective; *P*: relevant *P*-value. * indicates *p* < 0.05. ** indicates *p* < 0.01. *** indicates *p* < 0.001

Table 2 shows the mean scores for the ten items at baseline and follow-up. The results showed significant mean differences in seven out of ten items. Two out of four items of domain *T* were significant; two items out of three in domain 2 *R* were significant. In the third domain *O*, all three items were significant.

**Table 2:** Results of the pairwise t-test of the 10 SPICE items, *N* = 31

Item	M T0 (SD)	M T1 (SD)	*P* *
Working with another discipline of students enhances my education. **(T)**	4.55 (0.568)	4.74 (0.514)	*
Participating in educational experiences with another discipline of students enhances my future ability to work on an interdisciplinary team. **(T)**	4.61 (0.495)	4.58 (0.620)	0.823
All health professions students should be educated to establish collaborative relationships with members from other disciplines. **(T)**	4.84 (0.374)	4.90 (0.301)	0.325
During their education, medical and pharmacy students should be involved in teamwork in order to understand their respective roles. **(T)**	4.55 (0.675)	4.87 (0.341)	**
My role within the interdisciplinary team is clearly defined. **(R)**	3.16 (0.820)	3.84 (0.860)	***
I have an understanding of the courses taken by, and training requirements of, both pharmacy and medical students. **(R)**	3.48 (0.996)	3.52 (1.029)	0.879
I understand the roles of other professionals within the interdisciplinary team. **(R)**	3.55 (0.768)	4.29 (0.693)	***
Patient satisfaction is improved when patients are treated by a team of professionals from different disciplines. **(O)**	4.35 (0.839)	4.90 (0.301)	**
Healthcare costs are reduced when patients are treated by a team of professionals from different disciplines. **(O)**	3.74 (0.855)	4.23 (0.845)	**
Patient-centeredness increases when care is delivered by a team of professionals from different disciplines. **(O)**	4.13 (0.957)	4.58 (0.672)	**

The associated domain can be found in brackets: T = Interprofessional Teamwork and Team-based Practice; R = Roles/responsibilities for Collaborative Practice; O = Patient Outcomes from Collaborative Practice. The items are grouped within their domain but are numbered as in the questionnaire

M: mean score; SD: standard deviation; T0: baseline measurement; T1: measurement after completing the elective. P: *P*-value for the pairwise t-test. * indicates *p* < 0.05. ** indicates *p* < 0.01. *** indicates *p* < 0.001

No differences in the mean scores at baseline between medical and pharmacy students were found, nor were any differences in the mean scores found between male and female participants. There were no differences in scores in the three SPICE domains at baseline between participants who took part in both surveys and participants who only took part in the baseline survey.

## Discussion

This study aimed to evaluate whether an interprofessional educational intervention could lead to an improved attitude towards interprofessional collaboration. All three domains in the SPICE-2D questionnaire improved significantly, meaning that the students had a more positive attitude towards interprofessional education after they took part in the elective. Looking at the pre-post differences on item level we saw an improvement in most of the ten items. Results indicate that IPE had an impact on the perception of medical and pharmacy students regarding their views on and attitudes towards aspects of collaborative work like teamwork, responsibilities and outcomes for patients.

Linn et al. found that integrating a paired IPE model changed the perception of family medicine residents and pharmacy students within the team and that they valued the training requirements [2]. A fairly similar study with medical and pharmacy students observed that a multimodal and structured IPE approach had positive effects on students’ perception of “interprofessional clinical education” [3]. Comparing the results of our study with similar studies using the SPICE questionnaire, we noted that some studies found fewer significant results than we did [2–4]. Our study using the SPICE questionnaire confirms and extends these findings, possibly as a result of our relatively large sample size.

Sytsma et al. found that medical and physical therapy students showed an openness to IPE both at baseline and follow-up and they even showed this openness and a positive attitude towards IPE experience and collaborative work one year after baseline [11]. This means that although the intervention in this study was fairly short, it still had an impact even one year later. They also found significant differences in perception of roles and responsibilities between medical and physical therapy students. They also pointed out that the effects of multiple IPEs or lessons with a stronger focus on IPE are still unknown. Also worth noting, in this study, the participants were at the beginning of their training. In a systematic review, researchers Wang et al. found that IPE training improved understanding of collaboration and led to more positive attitudes towards teamwork [12]. Medical students as compared to dental students and female students as compared to male students tended to have more positive attitudes. The study suggests that IPE training has a positive impact on healthcare education, with sex playing an important role in the outcomes. In our study there were no differences between the medical and pharmaceutical students or in the students’ sex, regarding an improved attitude.

The final aim of IPE is improved IPC in later professional life, resulting in higher quality of care and more job satisfaction. An exploratory study Zechariah et al. indicated that medical students with or without prior experience in IPE valued the importance of both IPE and IPC [13]. These researchers also revealed a strong interest in attending courses that include IPE in other disciplines. In our study, we focused on IPE, but Walkenhorst et al. summarize in their position paper that IPE has the potential to improve IPC as well as patient outcomes [14].

A study using the Delphi method by Homeyer et al. indicates that in order to improve IPC and collaborative patient-centered care, IPE between medical and nursing staff should focus on interprofessional communication and roles understanding [15]. Other studies support the positive impact of IPE and IPC on treatment of patients and a successful working environment (e.g [13, 16]., while Reeves et al. pointed out that poor interprofessional collaboration can have a negative impact on patient care [17]. A systematic review by Ojelabi et al. points out positive outcomes for collaborative efforts in disease management and patient-centered healthcare, indicating positive improvements for diagnosis, treatment and quality of care [18]. Finally, Saragih et al. conclude that IPE enables students to develop their knowledge of healthcare [19].

For proper interpretation of our results, some strengths and limitations should be mentioned. Compared to other studies that included the SPICE questionnaire, our study had a fairly big sample size. Although the SPICE questionnaire was used in some prior studies, their focus was mostly validation of the questionnaire. Our procedure was more or less exploratory, meaning that our research setting and research focus had not previously been studied in depth and that we should interpret our significant results carefully. We did not perform an a priori sample size. Still, we were able to show statistically significant improvements, implying that our sample size was sufficient. Although we have to note that larger sample sizes generally improve the reliability and generalizability of study findings, recent methodological literature confirms that a sample size of 30 or more participants can provide sufficient power to detect significant differences in within-subject designs, such as ours. To reach generalizability of results future studies should not only have larger sample sizes; because of large and many differences in national care systems, studies should be performed in multiple countries.

Our sample consisted of pharmacy and medical students, representing a combination that is important due to their collaboration in later professional life. Because our elective is delivered by an epidemiologist, a clinical pharmacist together with a psychiatrist, a physician for clinical pharmacology, a pharmacist and an assistant medical director, we ensured interprofessional education from the teachers’ side as well.

The German version of the SPICE questionnaire used in this study was translated by the institute members, using a standardized method. Although our translated version was not validated, it was afterwards compared with a translated and validated questionnaire by Pudritz et al. [8], which was not known to us at the time we started our study. We noticed that this validated German questionnaire had very similarly formulated items, resulting in very few differences between it and our German SPICE questionnaire. Nevertheless, this might have impacted the reliability and comparability to other studies. Both German versions are available in Appendix 1. Looking at the reliability scores in our study, we see that they are somewhat similar to the calculated reliabilities in validation studies [8, 9]. The internal consistencies in our study were above 0.6 for all domains and domain 3 above 0.7. Compared to the validated German Version of the SPICE questionnaire, we had a lower internal consistency for domain 1, but clearly higher internal consistency for domains 2 and 3. Nevertheless, the results should be considered cautiously. In future studies, the addition of open-ended questions might provide more and in-depth insights in the learning experiences of the students and possibilities to improve.

Participants in our study, were students who chose to take part in the elective on the management of patients with multimorbidity and polypharmacy, with as one of the main aims participate in interprofessional learning. This might have resulted in more than averagely motivated student cohorts.

We had a considerable drop in response rate on surveys from baseline to follow-up. There were no differences between those only filling out baseline and those filling out both questionnaires, regarding sex, discipline (pharmacy or medicine), and baseline SPICE scores, implicating that our conclusions are robust. However, a selective non-response at follow-up, e.g. of students who were dissatisfied with the elective, cannot be excluded. In future studies, more attention could be paid to increase the follow-up response by providing the questionnaire online instead of paper-based only and by sending out reminders.

It is not clear whether the positive results are lasting, since we only have a follow-up survey shortly after completion of the elective. Further follow-up surveys, after e.g. six and 12 months, could shed light on this question. Moreover, the SPICE-2D questionnaire assesses the subjective view of the students’ *expectations* concerning the consequences of interprofessional collaboration on future quality and costs of care.

Looking at our study design, it must be stated that we did not have a control group. Guraya and Barr [20] also found that pre-post design studies on IPE showed a positive impact on students’ knowledge, skills, and attitudes, meaning that IPE has a positive effect on improving multiple aspects of collaborative interprofessional teamwork.

If larger studies confirm the positive effects of IPE on the topic of the management of multimorbidity and polypharmacy, the expansion of the IPE to other disciplines that play an important role in the care of those patients, such as nursing and physical therapy, is to be considered.

We conclude that the attitude towards IPE improves by integrating a standardized and interprofessional education into pharmacy and medical students’ studies. Although the results need to be reproduced using a different sample, one can assume that IPE contributes to more positive attitudes. We therefore recommend that interprofessional education for medical and pharmacy students be extended and include courses with students from other related studies.

## Data Availability

The datasets used and analyzed during the current study are available from the corresponding author on reasonable request.

## Abbreviations

α: Cronbach’s alpha
et al.: Et alia (and others)
IPC: Interprofessional Collaboration
IPE: Interprofessional Education
IPEC: Interprofessional Education Collaborative
IQR: Interquartile range
M: Mean Score
MD: Median
O: Patient Outcomes from Collaborative Practice
p: P-Value
R: Roles/responsibilities for Collaborative Practice
SD: Standard deviation
SPICE: Student Perceptions of Physician-Pharmacist Interprofessional Clinical Education
SPICE-2D: Student Perceptions of Physician-Pharmacist Interprofessional Clinical Education, Version 2, Deutsch
T: Interprofessional Teamwork and Team-based Practice
T0: Baseline measurement
T1: Measurement after completing the elective
WHO: World Health Organization
$$\:\stackrel{-}{x}$$: Mean score

## References

[1] A WHO report. framework for action on interprofessional education and collaborative practice - PubMed [Internet]. [cited 2024 Jan 9]. Available from: https://pubmed.ncbi.nlm.nih.gov/21174039/21174039

[2] Linn BS, Smith BEY, Cassel T. Impact of Collaborative Inpatient Pairing Between Pharmacy Students and Family Medicine Residents on Perceptions of Interprofessional Care. PRiMER [Internet]. 2022 May 25 [cited 2023 Sep 29];6. Available from: https://journals.stfm.org/primer/2022/linn-2021-0088/10.22454/PRiMER.2022.661338 PMC9256298 35801194

[3] Nwaesei AS, Jacob BC, Peasah SK, Perkins JJ, Hogan M. A structured approach to intentional interprofessional experiential education at a Non-Academic community hospital. Am J Pharm Educ. 2019;83(9):7365. 31871358 10.5688/ajpe7365 PMC6920644

[4] Zorek JA, MacLaughlin EJ, Fike DS, MacLaughlin AA, Samiuddin M, Young RB. Measuring changes in perception using the student perceptions of Physician-Pharmacist interprofessional clinical education (SPICE) instrument. BMC Med Educ. 2014;14(1):101. 24884800 10.1186/1472-6920-14-101 PMC4035824

[5] Alipour F, Peiravian F, Mehralian G. Perceptions, experiences and expectations of physicians regarding the role of pharmacists in low-income and middle-income countries: the case of Tehran hospital settings. BMJ Open. 2018;8(2):e019237. 10.1136/bmjopen-2017-019237 PMC5829860 29420231

[6] Zielińska-Tomczak Ł, Cerbin-Koczorowska M, Przymuszała P, Gałązka N, Marciniak R. Pharmacists’ perspectives on interprofessional collaboration with physicians in Poland: A quantitative study. Int J Environ Res Public Health. 2021;18(18):9686. 34574606 10.3390/ijerph18189686 PMC8470388

[7] Fike DS, Zorek JA, MacLaughlin AA, Samiuddin M, Young RB, MacLaughlin EJ. Development and validation of the student perceptions of physician-pharmacist interprofessional clinical education (SPICE) instrument. Am J Pharm Educ. 2013;77(9):190. 24249852 10.5688/ajpe779190 PMC3831401

[8] Pudritz YM, Fischer MR, Eickhoff JC, Zorek JA. Validity and reliability of an adapted German version of the student perceptions of Physician-Pharmacist interprofessional clinical education instrument, version 2 (SPICE-2D). Int J Pharm Pract. 2020;28(2):142–9. 31373100 10.1111/ijpp.12568

[9] Zorek JA, Fike DS, Eickhoff JC, Engle JA, MacLaughlin EJ, Dominguez DG, et al. Refinement and validation of the student perceptions of Physician-Pharmacist interprofessional clinical education instrument. Am J Pharm Educ. 2016;80(3):47. 27170818 10.5688/ajpe80347 PMC4857642

[10] Eremenco SL, Cella D, Arnold BJ. A comprehensive method for the translation and cross-cultural validation of health status questionnaires. Eval Health Prof. 2005;28(2):212–32. 15851774 10.1177/0163278705275342

[11] Sytsma TT, Haller EP, Youdas JW, Krause DA, Hellyer NJ, Pawlina W, et al. Long-term effect of a short interprofessional education interaction between medical and physical therapy students. Anat Sci Educ. 2015;8(4):317–23. 26040635 10.1002/ase.1546

[12] Wang Z, Feng F, Gao S, Yang J. A systematic Meta-Analysis of the effect of interprofessional education on health professions students’ attitudes. J Dent Educ. 2019;83(12):1361–9. 31548305 10.21815/JDE.019.147

[13] Zechariah S, Ansa BE, Johnson SW, Gates AM, Leo GD. Interprofessional education and collaboration in healthcare: an exploratory study of the perspectives of medical students in the united States. Healthcare. 2019;7(4):117. 31618920 10.3390/healthcare7040117 PMC6956332

[14] Walkenhorst U, Mahler C, Aistleithner R, Hahn EG, Kaap-Fröhlich S, Karstens S et al. Position statement GMA Comittee– Interprofessional Education for the Health Care Professions. 2015;32. 10.3205/zma000964 PMC4446653 26038687

[15] Homeyer S, Hoffmann W, Hingst P, Oppermann RF, Dreier-Wolfgramm A. Effects of interprofessional education for medical and nursing students: enablers, barriers and expectations for optimizing future interprofessional collaboration– a qualitative study. BMC Nurs. 2018;17:13. 29643742 10.1186/s12912-018-0279-x PMC5891914

[16] Kaiser L, Conrad S, Neugebauer EAM, Pietsch B, Pieper D. Interprofessional collaboration and patient-reported outcomes in inpatient care: a systematic review. Syst Rev. 2022;11(1):169. 35964148 10.1186/s13643-022-02027-x PMC9375378

[17] Reeves S, Pelone F, Harrison R, Goldman J, Zwarenstein M. Interprofessional collaboration to improve professional practice and healthcare outcomes. Cochrane Database Syst Rev [Internet]. 2017 [cited 2024 Feb 3];(6). Available from: https://www.cochranelibrary.com/cdsr/doi/10.1002/14651858.CD000072.pub3/full 10.1002/14651858.CD000072.pub3 PMC6481564 28639262

[18] Ojelabi AO, Ling J, Roberts D, Hawkins C. Does interprofessional education support integration of care services? A systematic review. J Interprofessional Educ Pract. 2022;28:100534.

[19] Saragih ID, Arna Uly Tarihoran DET, Sharma S, Chou FH. A systematic review and meta-analysis of outcomes of interprofessional education for healthcare students from seven countries. Nurse Educ Pract. 2023;71:103683. 37433234 10.1016/j.nepr.2023.103683

[20] Guraya SY, Barr H. The effectiveness of interprofessional education in healthcare: A systematic review and meta-analysis. Kaohsiung J Med Sci. 2018;34(3):160–5. 29475463 10.1016/j.kjms.2017.12.009 PMC12977169

